# *clc *is co-expressed with *clf *or *cntfr *in developing mouse muscles

**DOI:** 10.1186/1478-811X-3-1

**Published:** 2005-01-31

**Authors:** Béatrice de Bovis, Damien Derouet, Jean-François Gauchat, Greg Elson, Hugues Gascan, Odile deLapeyrière

**Affiliations:** 1 INSERM UMR 623, Developmental Biology Institute of Marseille (CNRS – INSERM – Univ. Méditerranée), Campus de Luminy, Case 907, 13288 Marseille Cedex 09, France; 2 INSERM U564, CHU d'Angers, 4 rue Larrey, 49033 Angers Cedex, France; 3 Département de Pharmacologie, Université de Montréal, 2900 Édouard-Montpetit, Montréal QC H3T 1J4, Canada; 4 NovImmune SA, 1211 Geneva, Switzerland

## Abstract

**Background:**

The ciliary neurotrophic factor (CNTF) receptor is composed of two signalling receptor chains, gp130 and the leukaemia inhibitory factor receptor, associated with a non-signalling CNTF binding receptor α component (CNTFR). This tripartite receptor has been shown to play important roles in the development of motor neurons, but the identity of the relevant ligand(s) is still not clearly established. Recently, we have identified two new ligands for the CNTF receptor complex. These are heterodimeric cytokines composed of cardiotrophin-like cytokine (CLC) associated either with the soluble receptor subunit cytokine-like factor-1 (CLF) or the soluble form of the binding receptor itself (sCNTFR).

**Results:**

Here we show that, during development, *clc *is expressed in lung, kidney, vibrissae, tooth, epithelia and muscles during the period of development corresponding to when motoneuron loss is observed in mice lacking a functional CNTF receptor. In addition, we demonstrate that it is co-expressed at the single cell level with *clf *and *cntfr*, supporting the idea that CLC might be co-secreted with either CLF or sCNTFR.

**Conclusion:**

This expression pattern is in favor of CLC, associated either with CLF or sCNTFR, being an important player in the signal triggered by the CNTF receptor being required for motoneuron development.

## Background

CLC (cardiotrophin-like cytokine) shares homology with CNTF (ciliary neurotrophic factor) and CT-1 (cardiotrophin-1) and requires co-expression with either CLF (cytokine-like factor-1) or the soluble form of the CNTFR to be secreted [1,2]. The CLC-CLF heterodimer displays activities only on cells expressing a functional CNTF receptor [1] and therefore CLC is likely to be part of the developmentally important second ligand for CNTFR. The existence of such a second ligand has been suggested by the phenotype of mice lacking any of the three receptor subunits comprising the functional CNTF receptor complex (LIFRβ, gp130 and CNTFR) which exhibit significant reductions in motoneuron number [3-5] whereas CNTF-deficient mice have no motoneuron loss during development [6]. There are however two prerequisites for CLC to play a major role in motoneuron development: 1) CLC must be expressed in the environment of motoneurons during development. 2) As it cannot be secreted alone, it must be co-expressed with either CLF or sCNTFR, in the same cell.

## Results and Discussion

### Developmental expression of clc

Since the expression of *clc *has only been studied in adult mouse tissues [7], we first examined the expression of genes encoding CLC or its co-secreted proteins, CLF and CNTFR in various embryonic tissues using reverse transcription and quantitative real-time polymerase chain reaction (RT-PCR). In all tissues tested from E16.5 and E18.5 (Table 1), the level of expression of *clc *is very low when compared with that of *clf *or *cntfr*. The highest level of *clc *expression was observed in the muzzle, a very heterogeneous region containing different positive tissues, as described below. *Clc *expression is also observed in lung, kidney, brain and skeletal muscles such as the tongue or limb muscles.

**Table 1:** RT-PCR analysis of *clc*, *clf *and *cntfr *expression^a^

	***clc***		***clf***		***cntfr***	
	
	**E16.5**	**E18.5**	**E16.5**	**E18.5**	**E16.5**	**E18.5**
Skeletal muscle	0.113 ± 0.02	0.936 ± 0.12	0.232 ± 0.003	24.84 ± 2.13	90.25 ± 2.71	6.72 ± 0.76
Heart	NS^c^	NS	NS	NS	NS	NS
Tongue	3.12 ± 0.12	1.2 ± 0.09	495 ± 17.1	77.1 ± 4.64	28.3 ± 10.9	2.6 ± 0.83
Muzzle	11.4 ± 0.75	9.25 ± 0.79	1050 ± 65.3	524 ± 85.8	425 ± 47.5	57.9 ± 4.02
Lung	4.08 ± 0.65	16.4 ± 0.48	2290 ± 490	2240 ± 184	43.8 ± 15.6	ND^b^
Kidney	5.55 ± 0.12	6.75 ± 1.15	61.4 ± 4.59	ND	51.8 ± 9.49	19.8 ± 3.12
Liver	0.191 ± 0.02	0.129 ± 0.03	0.071 ± 0.001	0.034 ± 0.09	0.047 ± 0.002	0.038 ± 0.03
Brain	1.04 ± 0.12	0.317 ± 0.09	150 ± 3.39	74.3 ± 16.4	216 ± 2.01	236 ± 16.1
Spinal cord	ND	0.183 ± 0.01	0.123 ± 0.01	61.2 ± 6.25	6.13 ± 0.03	109 ± 27.4

^a^Expression of *clc*, *clf *and *cntfr *was determined using reverse transcription and quantitative real-time PCR as detailed in Experimental Procedures and expressed as fM of cDNA/μg total RNA.

^b^not determined

^c^not significant

**Figure 1:**
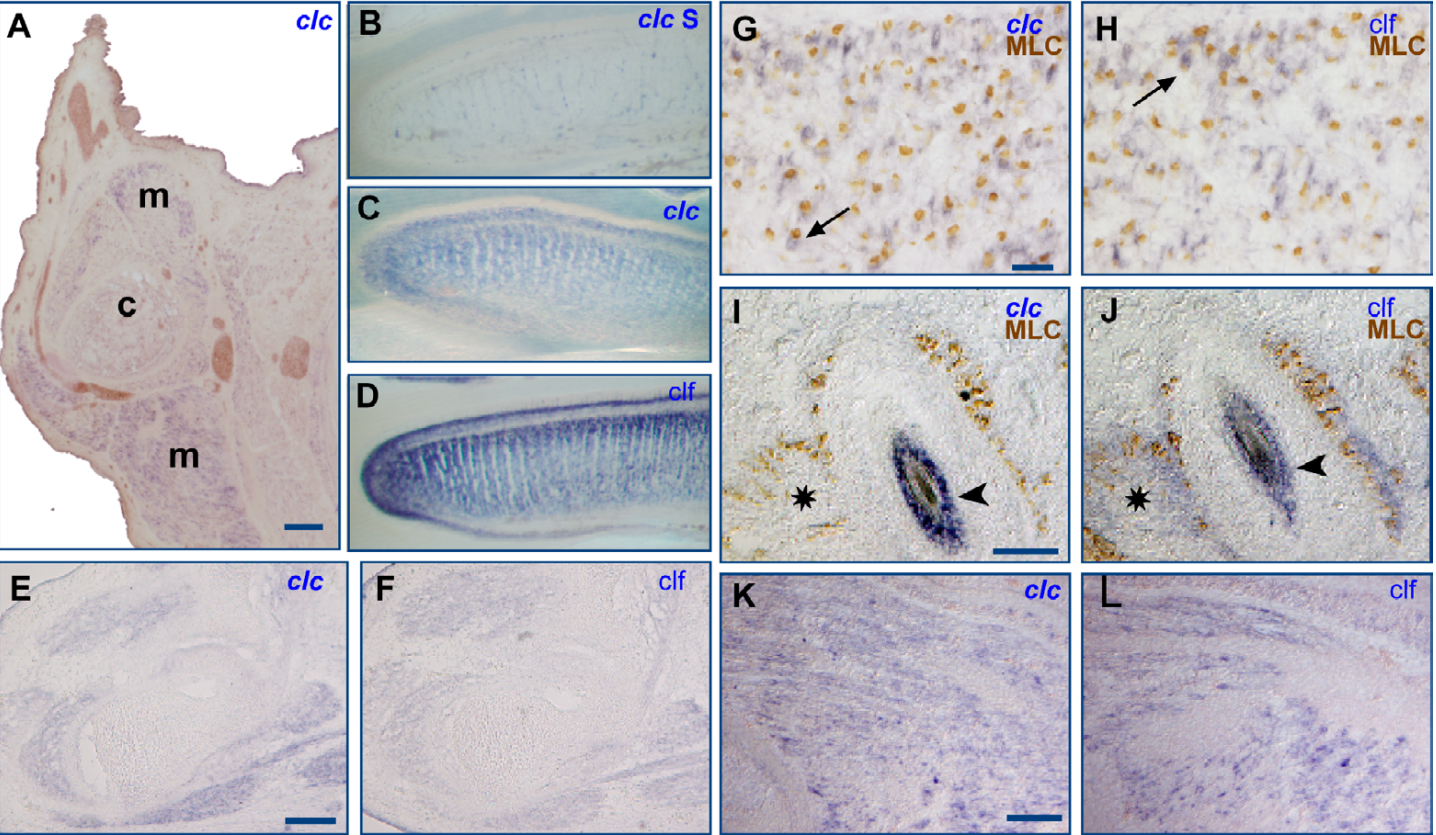
**In E16.5 mouse embryos *clc *is expressed in muscles. **Cryostat sections (A, E-L) or vibratome sections (B-D) from E16.5 (A-J) or E14.5 (K, L) were hybridized to *clc *(A, C, E, G, I and K) or *clf *(D, F, H, J and L). The control *clc *sense probe gave rise to very faint staining (B). Transverse section through the forelimb (A, K and L), the hindlimb (E and F) and saggital sections through the tongue (C and D) showing expression of *clc *and *clf *in muscles. The identity of the *clc*-positive cells such as muscle fibers was confirmed by double staining and compared to *clf*-positive cells. *In situ *hybridization using Dig-labeled probes for *clc *and *clf *(cytoplasmic blue staining) was performed on sections through shoulder muscles (G, H) or vibrissae (I, J) from E16.5 MLC*nlacZ *mice, which express the *nlacZ *reporter gene under the control of a muscle-specific MLC promoter. Subsequently, the sections were processed for immunohistochemical detection of β-galactosidase (nuclear brown staining). Arrows indicate double-labeled cells. Transverse section through the muzzle (I and J) shows that vibrissae (arrowheads) are positive for *clc *and *clf *whereas only *clf *is detected in muscles (asterisks) surrounding vibrissae. c, cartilage; m, muscle. Scale bars are 200 μm in A, E and F, 25 μm in G and H and 100 μm in I-L.

To further assess the potential involvement of CLC in the development of motoneurons, we performed *in situ *hybridization experiments to determine the pattern of expression of *clc *in the environment of developing motoneurons and compare it with the expression of both *clf *and *cntfr*. Motoneuron death occurs between E14.5 and E18.5 in mice lacking in the ability to produce a functional CNTF receptor complex [5], suggesting that expression of CNTFR and its relevant ligands is critical between these timepoints. We therefore studied *clc *mRNA expression levels at E16.5. *Clc *is expressed in muscles along the whole rostro-caudal axis, at the brachial level (Fig. 1A) as well as at the lumbar level (Fig. 1E and [8]. It is also expressed in the tongue (Fig. 1C) like clf (Fig. 1D). The identity of muscle cells (Fig. 1G) was confirmed by double staining performed on transgenic mice with the *nlacZ *reporter gene under the control of the muscle-specific MLC promoter [9]. All *clc*-positive muscle fibers also stained positive for *clf *(Fig. 1E, 1F, 1G, 1H and [8]). *clc *expression was not detected in certain *clf*-positive muscles however, such those around the vibrissae (Fig. 1I and 1J). Since the level of *clc *expression is generally low, this could reflect the limited sensitivity of the *in situ *hybridization technique used. To determine the onset of *clc *and *clf *expression in the muscles, the motoneuron targets, we performed in situ hybridizations at different stages. *Clc *and *clf *are expressed, although at low levels, as soon as the muscles develop and are clearly observed at E14.5 (Fig. 1K and 1L).

**Figure 2:**
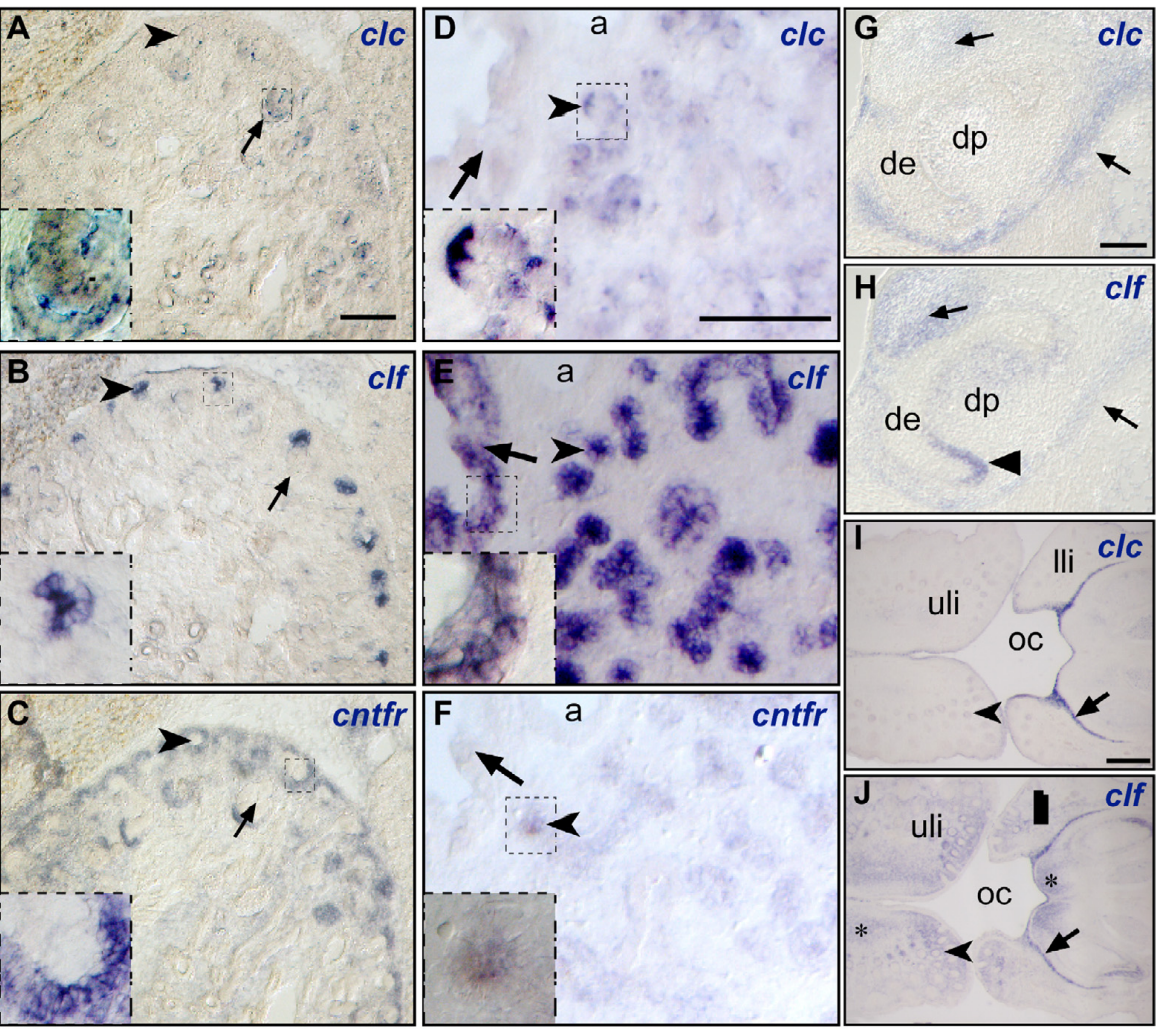
***Clc *is expressed in epithelia **Transverse sections from E16.5 mouse embryos were hybridized to *clc *(A, D, G and I), *clf *(B, E, H and J) or *cntfr *(C and F). Sections through the kidney (A-C) show that *clc *is expressed in developing nephrons (arrows), *clf *in ureteric tips (arrowheads) and *cntfr *in nephrogenic mesenchyme. Sections through the lung (D-F) show that whereas *clf *is strongly expressed in both distal (arrowheads) and proximal (arrows) epithelia, *clc *and *cntfr *are weakly expressed in distal epithelium. Boxed areas are shown in higher magnification in the corner of each panel. Sections through molar tooth germs (G, H) show that mesenchyma (arrows) surrounding the dental follicle is positive for both *clc *and *clf *and that the inner enamel epithelium (arrowheads) expresses only *clf*. Coronal sections through muzzle (I, J) show that both *clc *and *clf *are expressed in the epithelium bordering the mandibles and in between the lips and mandibles (arrow) as well as in follicles of vibrissae (arrowheads); in addition, *clf *is expressed in mesenchyma (asterisks). a, pulmonary artery; dp, dental papilla; de, dental epithelium; oc, oral cavity; uli, upper lip; lli, lower lip. Bars: 100 μm in A-H, 200 μm in I and J.

*Clc *is also expressed in several organs in which reciprocal epithelial-mesenchymal interactions are essential, such as the developing vibrissae (Fig. 1I and 2I), tooth, kidney, and lung. In the kidney, *clc *is expressed in the comma-shaped body (Fig. 2A). Strikingly, CLF and CNTFR are expressed in different structures, *clf *being synthesized in the tips of the ureteric (Fig. 2B) and *cntfr *being synthesized by mesenchyma cells surrounding these structures (Fig. 2C). In the lung, both *clc *and *cnftr *are expressed faintly in distal airway epithelium whereas *clf *is strongly expressed in distal and proximal epithelia (Fig. 2D, 2E and 2F). Sections through molar tooth germs (Fig. 2G and 2H) show that *clf *is expressed in both the mesenchyma surrounding the dental follicle which gives rise to alveolar bone and the inner enamel epithelium whereas *clc *is expressed only in the former. *Clc *and *clf *are also co-expressed in the epithelium bordering the mandibles and the lips although *clf *is also expressed in mesenchyma (Fig. 2I and 2J). Together these results are in agreement with the expression pattern described for both *clf *[10] and *cntfr *[11].

### Co-expression of *clc*, *clf *and *cntfr *in the developing muscle

**Figure 3:**
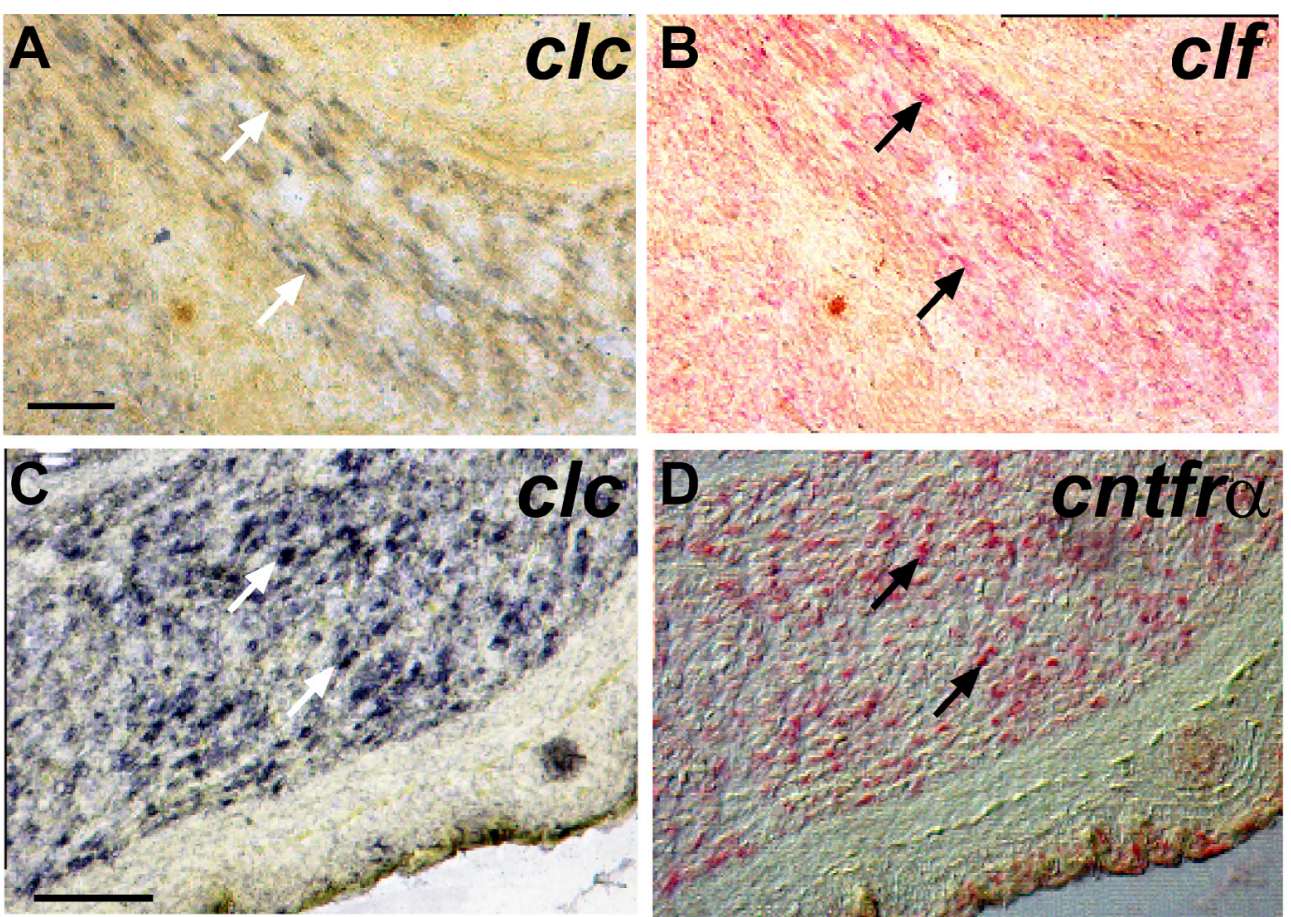
**Double-labeling detects co-expression of *clc *and *clf *or *clc *and *cntfr *in individual muscle cells. **Single sections of E16.5 muscles were hybridized with two probes. Dig-labeled *clc *(A-D) and Fluo-labeled *clf *(A, B) or *cntfr *(C, D). Anti-Dig antibodies were applied first and stained using NBT/BCIP to reveal cells expressing *clc *(A, C). Anti-Fluo antibodies were then applied and detected using Fast red to reveal cells expressing *clf *(B) and *cntfr *(D), after removal of the first red reaction product. Most muscle cells express *clc *and *clf *or *clc *and *cntfr *(examples indicated by arrows). Bars: 100 μm.

In transfected cells CLC requires either CLF or sCNTFR to be secreted [1,2]. This cooperative effect requires the expression of genes for both factors in the same cell. To ascertain whether a single muscle cell can express at least CLC and CLF or CLC and sCNTFR, we studied co-expression on hind-limb muscle sections. We performed double *in situ *hybridization of *clc *and *clf *and of *clc *and *cntfr*. Most muscle cells expressed both *clc*, (revealed using NBT/BCIP; Fig. 3A, C) and *clf *or *cntfr *(Fig. 3B, D; revealed using Fast Red). Co-expression was observed at the single cell level demonstrating that *in vivo *CLC could be co-secreted either with CLF or sCNTFR.

## Conclusions

*Clc *is expressed in developing muscles during the period of motoneuron loss in mice lacking a functional CNTF receptor and it is co-expressed with both CLF and CNTFR. This expression pattern is in favor of the hypothesis that CLC is an important player in the signal triggered by the CNTF receptor and that is required for motoneuron development. In addition, our results show that in the kidney, *clc *is expressed in cells neighboring those expressing *clf *or *cntfr *but it is not co-expressed with these genes suggesting either the possible existence of an additional protein capable of inducing secretion of CLC or that CLC is not secreted in these cells and therefore not functional. Because genetic deletion of *cntf *fails to perturb neuronal development before birth, we can hypothesize some functional redundancies *in vivo *that will require the analysis of double or triple knockout mice for CNTFR ligands to clarify their respective involvement in mouse neural development.

## Methods

### RT and real time PCR

Total RNA was extracted using Trizol reagent (Invitrogen) from E16.5 or E18.5 mouse tissues according to the manufacturer's instructions. Complementary cDNA was synthesised from 2 μg of RNA by random hexamer priming using MMLV reverse transcriptase (Promega). Quantitative PCR was performed using a capillary real-time LightCycler (Roche Diagnostics), and the data analysed using "Fit Point Method" (Roche Diagnostics). For comparison of gene expression levels, all quantifications were normalized to endogenous *gapdh *to account for variability in the initial concentration of RNA and for differences in the efficiency of the reverse transcription reactions. The following primers were designed to amplify mouse *clc*: 5'-GCTACCTGGAGCATCAACT-3', 5'-GGTGACTGTACGCCTCATAG-3'; *clf*: 5'-CAGTCAGGAGACAATCTGGT-3', 5'-ACGTGAGATCCTTCATGTTC-3'; *cntfr*: 5'-CTACATCCCCAATACCTACA-3', 5'-GTGAATTCGTCAAAGGTGAT-3'; *gapdh*: 5'-TGCGACTTCAACAGCAACTC-3', 5'-CTTGCTCAGTGTCCTTGCTG-3'. Results are expressed in fmole of cDNA/μgRNA.

### Probes

Plasmid cDNA clones were linearized and transcribed with T7 or T3 polymerase using digoxigenin (Dig) or fluorescein (Fluo)labeling reagents (Roche Diagnostics). Probes were used at a concentration of 500 ng/ml. The *cntfr *clone was as previously described [12] and the mouse *clf *[13] and *clc *probes corresponded to the isolated cDNAs.

### In situ hybridization

*In situ *hybridization was performed as described previously [14] on 20 μm-thick frozen transverse cryostat sections prepared from mouse embryos fixed with 4% paraformaldehyde in PBS, and cryopreserved in 15% sucrose in PBS before embedding in OCT compound (Miles). Alternatively, 100 μm-thick vibratome sections were prepared from fixed embryos embedded in glutaraldehyde/gelatin. After hybridization overnight at 70°C with Dig-labeled riboprobes, the slides were washed twice in 1X SSC, 50% formamide at 70°C for 30 min and blocked in the presence of 4% blocking reagent (Roche Diagnostics) and 20% inactivated sheep serum. The slides were then incubated with anti-Dig-alkaline-phosphatase (AP)-conjugated antibody (1/5000, Roche Diagnostics), washed and revealed by NBT/BCIP staining.

In order to confirm that muscle fibers, *per se*, express *clc *and *clf*, double *in situ *hybridization / immunohistochemistry was carried out as described [15] on sections from E16.5 MLC*nlacZ *mice, which express the *nlacZ *reporter gene under the control of a muscle-specific myosin light chain promoter. After *in situ *hybridization, slides were rinsed in PBT (PBS, 0.1% Triton), and sections were successively incubated for 1 h with blocking solution containing 2% BSA, 2% heat-inactivated donkey serum in PBT and then overnight at 4°C with rabbit anti-β-galactosidasel (1/1000, Cappel). After three washes in PBT, slides were incubated 1 h at RT with a biotin donkey anti-mouse secondary antibody. Slides were then washed in PBS, and TBS (50 mM Tris-HCl, 0.15 M NaCl, pH 7.6), and incubated for 30 min at RT in ABC streptavidin/HRP in TBS. Staining was revealed with DAB (D4293, Sigma) in the presence of H_2_O_2_.

Double *in situ *hybridization was performed as described previously [14]. Briefly, Dig- and Fluo-labeled probes were mixed in hybridization buffer and applied to sections. After hybridization at 70°C overnight and washing at 65°C, the first probe was revealed using a 1:2000 dilution of anti-Fluo-alkaline phosphatase (AP)- conjugate (Roche Diagnostics) and Fast Red (Sigma) as a substrate. Sections were photographed at this stage. After AP inactivation with 0.1 M glycine, pH 2.2, the second probe was revealed using a 1:5000 dilution of anti-Dig-AP and NBT/BCIP staining. Fast Red precipitates were then removed by incubating the slides in increasing concentrations of ethanol culminating in two final incubations in 100% ethanol for 10 min before cleaning with Histoclear and mounting with Eukitt (VWR, Strasbourg, France). Photomicrographs of the NBT/BCIP results were then taken for comparison with those showing the Fast Red results on the same sections.

## Competing interests

The author(s) declare that they have no competing interests.

## Authors' contributions

BB performed in situ hybridizations whereas DD and HG performed RT-PCR analyses. GE and JFG provided the *clc *and *clf *probes before publication. OL participated in the experimental design and coordination of the research. All authors read and approved the final manuscript.
